# Annual Dynamics of Mycobiota in Symptomatic Century-Old Trees of *Aesculus hippocastanum*, *Fagus sylvatica*, *Populus alba*, and *Quercus robur*

**DOI:** 10.3390/jof12010050

**Published:** 2026-01-11

**Authors:** Milan Spetik, Lucie Frejlichova, Jana Cechova, Pavel Bulir, Lenka Miksova, Lukas Stefl, Pavel Simek, Ales Eichmeier

**Affiliations:** 1 Mendeleum–Institute of Genetics, Mendel University in Brno, Valticka 334, 691 44 Lednice, Czech Republic; 2 Department of Planting Design and Maintenance, Mendel University in Brno, Valticka 334, 691 44 Lednice, Czech Republic

**Keywords:** co-occurrence networks, fungal diversity, fungal succession, high-throughput amplicon sequencing, host specificity, latent pathogens, tree mycobiome, trophic guilds, wood-inhabiting fungi

## Abstract

This study investigated the composition and temporal dynamics of wood-inhabiting fungal communities in four aging tree species in Lednice Castle Park (Czech Republic), located within the Lednice–Valtice Cultural Landscape, a UNESCO World Heritage Site. Forty wood cores were collected from 20 trees at two time points (2023 and 2024). The hosts included horse chestnut (*Aesculus hippocastanum* L.), copper beech (*Fagus sylvatica* ‘Atropunicea’ L.), oak (*Quercus robur* L.), and poplar (*Populus alba* L.), each exhibiting visual signs of decline. Fungal assemblages were profiled using ITS2 high-throughput amplicon sequencing. Ascomycota dominated across all hosts (72–89% of reads), while Basidiomycota contributed 8–24%, largely represented by Agaricomycetes in *F. sylvatica*. Alpha diversity varied significantly among hosts (Shannon: *F*_3,36_ = 10.61, *p* = 0.001 in 2023; *F*_3,36_ = 10.00, *p* = 0.001 in 2024). Temporal shifts were host-dependent: *F. sylvatica* exhibited the strongest year-to-year decline in richness (Chao1: −83%, *p* = 0.007) and increased beta dispersion, while *A. hippocastanum* and *P. alba* showed significant increases in diversity (+65% and +42%, respectively). Community composition was shaped by host species (PERMANOVA Bray–Curtis: *p* = 0.001) and shifted over time (Jaccard: *p* = 0.001), with *F. sylvatica* showing the highest temporal turnover. Functional guild analysis revealed consistent dominance of saprotrophs (29–41%) and mixed pathotroph–saprotroph guilds (23–36%) across hosts, indicating active degradation processes inside functional xylem. These results indicate that, within the studied system, the wood mycobiome of aging trees is host-dependent and temporally dynamic rather than static or functionally neutral. Short-term temporal turnover observed between sampling years may contribute to shifts in fungal community composition and succession within wood, with potential implications for tree decline processes in managed historical park landscapes.

## 1. Introduction

Old trees, particularly those exceeding a century in age, are vital components of park landscapes, contributing significantly to the ecological, aesthetic, and cultural integrity of these environments [1,2]. These veteran trees enhance landscape architectural compositions by providing visual coherence, historical authenticity, and functional benefits, ranging from fine-scale design elements to large-scale garden art features [3]. However, their advanced age renders them increasingly vulnerable to structural weaknesses and pathogen-induced decline, threatening their longevity and ecosystem services [4]. Understanding the intrinsic properties and associated vulnerabilities of these trees is therefore critical for their conservation and sustainable management [5].

Traditional methods for assessing tree health, such as visual inspections, often detect pathological issues only in advanced stages, when external symptoms like canopy dieback or basidiomycete fruiting bodies become evident, indicating significant decay or disease progression [6,7]. These approaches are limited in detecting early-stage or cryptic infections and fail to directly identify fungi within wood, which are often the primary cause of tree diseases [8,9,10]. This delay in diagnosis underscores the need for advanced molecular techniques to enable timely intervention and support effective conservation and management of trees [11,12].

Fungal communities inhabiting trees encompass a broad spectrum of endophytic and pathogenic taxa that influence host health [13,14,15], wood decay [16], and overall ecosystem functioning [17]. Wood-inhabiting fungi can occur as mycelia or spores and have traditionally been detected by isolation and culture-based identification [18]. However, these cultivation-based methods often underestimate fungal diversity because they typically favor fast-growing or spore-forming taxa and may miss unculturable or rare species [19,20,21]. To overcome these limitations, molecular methods, such as metabarcoding, have become essential [22]. By applying high-throughput sequencing of specific DNA regions, metabarcoding enables detection of a broader taxonomic range, including unculturable and previously undescribed taxa [23,24].

Although the importance of tree-associated mycobiota is increasingly recognized [25], detailed knowledge of their composition and temporal dynamics in managed historical park landscapes remains limited. A recent single-tree study of the trunk wood mycobiome of an ancient *Tilia × europaea* L. in the Czech Republic demonstrated that even individual heritage trees can host diverse and functionally complex fungal assemblages [26]. Environmental stressors [27], disease pressure [28], and anthropogenic impacts [29,30] may alter fungal assemblages, yet few studies have investigated their temporal dynamics at the level of individual trees [31,32,33]. To address this gap, we investigated the wood-associated fungal microbiome of 20 trees with an average age of ~100 years, representing four species—horse chestnut (*Aesculus hippocastanum* L.), copper beech (*Fagus sylvatica* ‘Atropunicea’ L.), pedunculate oak (*Quercus robur* L.), and white poplar (*Populus alba* L.)—in the Lednice Castle Park, part of the Lednice–Valtice Cultural Landscape listed as a UNESCO World Heritage Site. Importantly, destructive wood-core sampling of healthy monumental or near-monumental trees is heavily restricted by the Czech State Heritage Institute and the park administration. Permission for the present study was therefore granted exclusively for declining veteran trees that are part of the official long-term monitoring programme. Consequently, the study focuses on comparisons among tree species and temporal dynamics under comparable age-related decline conditions, rather than contrasts between healthy and declining individuals.

Using high-throughput amplicon sequencing (HTAS), we addressed three key questions: (i) how species richness and community composition differ among tree hosts, (ii) to what extent fungal communities remain stable over an annual timescale, and (iii) how temporal shifts in fungal community composition and predicted functional guilds differ among tree species exhibiting comparable visible symptoms of age-related decline. Our findings provide new insights into the stability and variability of wood-associated mycobiota in old heritage trees and highlight their potential role as bioindicators for the management of historical park landscapes.

## 2. Materials and Methods

### 2.1. Wood Sampling and Processing

**Figure 1:**
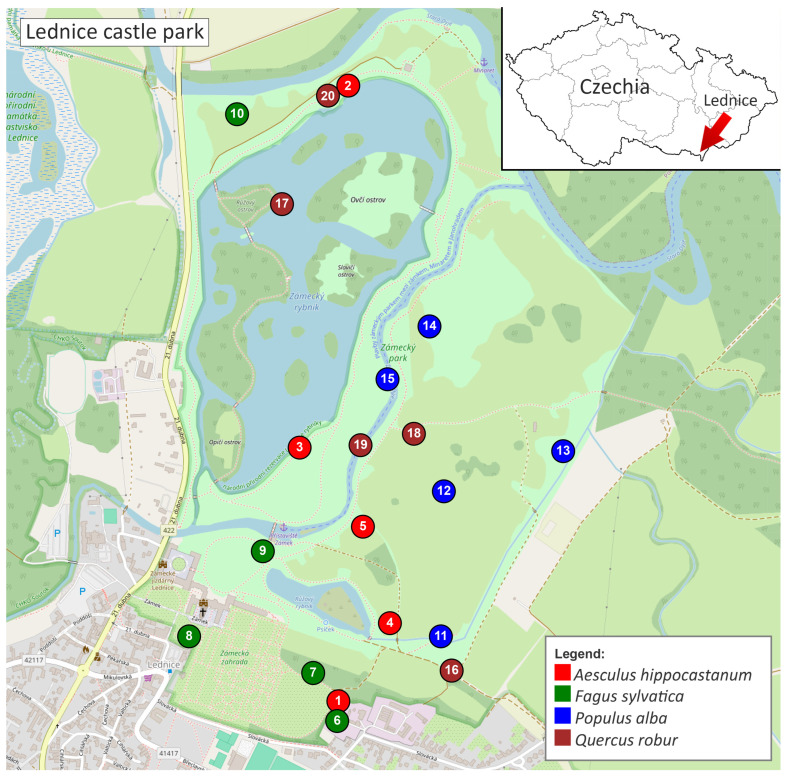
Map of the Lednice Castle Park showing the locations of sampled trees. Colors indicate tree species: *Aesculus hippocastanum* (red), *Fagus sylvatica* ‘Atropunicea’ (green), *Populus alba* (blue), and *Quercus robur* (brown). Numbers refer to tree IDs listed in Table 1. The map was created based on data ^©^ OpenStreetMap contributors. An interactive version of the map is available as Supplementary File S1.

**Figure 2:**
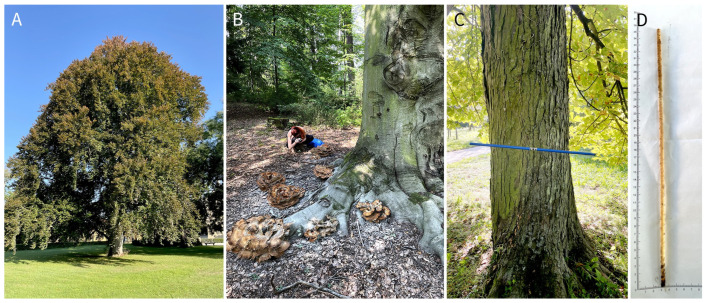
Field sampling and examples of tree conditions included in this study. (**A**) *Fagus sylvatica* ‘Atropunicea’ representing a typical sampled tree in the Lednice Castle Park. (**B**) *Fagus sylvatica* ‘Atropunicea’ with multiple basidiomata of *Meripilus giganteus* present at the base of the trunk. (**C**) *Aesculus hippocastanum* sampled for wood cores using an increment borer. (**D**) Example of an increment core extracted from *Fagus sylvatica* ‘Atropunicea’.

Wood cores were collected from 20 mature trees (File S1; Figure S1; Table 1), in Lednice Castle Park, Czech Republic (Figure 1). These trees represent four host species (*Aesculus hippocastanum*, *Fagus sylvatica* ‘Atropunicea’, *Quercus robur* and *Populus alba*, *n* = 5 trees per species). Trees (Figure 2) were selected based on external symptoms of decline (crown dieback, cankers, exudates), ensuring inclusion of individuals potentially affected by internal wood-colonizing fungi while avoiding dead or structurally unstable trees. Sampling was conducted at two time points (October 2023 and October 2024) to assess temporal dynamics, yielding a total of 120 wood samples (20 trees × 3 cores × 2 years).

Wood cores were obtained at breast height (1.3 m above ground) using a sterilized increment borer (80 cm, 5 mm diameter), sampling from three opposing trunk positions per tree to reduce spatial bias. Bark was disinfected with 70% ethanol prior to drilling to minimize contamination. After each core, the increment borer was disinfected in 5% sodium hypochlorite for 1 min and rinsed three times in sterile distilled water. Cores were individually wrapped in sterile Parafilm^©^, transported on ice, and stored at −20 °C until DNA extraction. In the laboratory, the inner sterile portions of wood cores were scraped and pooled per tree, homogenized under liquid nitrogen and processed for fungal DNA isolation.

### 2.2. Extraction of the DNA and DNA Pooling

Approximately 100 mg of homogenized inner wood tissue was used for DNA extraction. The sample was processed using the NucleoSpin^®^ Tissue Kit (Macherey-Nagel, Düren, Germany) following the manufacturer’s instructions. The total DNA yield was quantified using a fluorimeter and subsequently diluted to 10 ng·μL^−1^ for downstream molecular analyses.

### 2.3. Library Preparation and Sequencing

The fungal ITS2 region was amplified using the barcoded primer pair fITS7 and ITS4 [34,35]. PCR reactions were performed in 50 µL volumes containing 25 µL Q5^®^ High-Fidelity 2 × Master Mix (New England Biolabs, Ipswich, MA, USA), 2.5 µL of each primer (10 µM), 2 µL of template DNA (10 ng µL^−1^) and 18 µL nuclease-free water. The thermocycling protocol consisted of an initial denaturation at 95 °C for 2 min, followed by 35 cycles of 95 °C for 30 s, 55 °C for 30 s and 72 °C for 60 s, and a final extension at 72 °C for 5 min.

Amplicons were visualized on 1.2% agarose gels (Serva, Heidelberg, Germany) and purified using the NucleoSpin^®^ Gel and PCR Clean-up Kit (Macherey-Nagel, Düren, Germany). Indexed paired-end libraries were prepared with the Illumina Nextera XT DNA Library Preparation Kit (Illumina, San Diego, CA, USA) following the manufacturer’s protocol. Library quality and fragment size distribution were verified using a Fast qPCR Library Quantification Kit (MCLAB, San Francisco, CA, USA). Sequencing was carried out on an Illumina MiniSeq platform using 2 × 150 bp paired-end chemistry (MiniSeq Mid Output Kit(Illumina, Inc., San Diego, CA, USA), 300 cycles; Illumina, Inc., San Diego, CA, USA).

Negative control was included during DNA extraction, PCR amplification and sequencing to monitor potential contamination, and a mock fungal community was used as a positive control to validate amplification and sequencing performance. A mock community was prepared in situ using six fungal isolates relevant to the study system. The isolates included *Jattaea* sp. (MEND-F-0115), *Penicillium subericola* (MEND-F-1224), *Ilyonectria leucospermi* (MEND-F-1185), *Neofusicoccum parvum* (MEND-F-1155), *Trichoderma paratroviride* (MEND-F-0012), and *Diplodia seriata* (MEND-F-1195). Genomic DNA from these isolates was combined in defined proportions (5%, 15%, 15%, 10%, 25%, and 30%, respectively), and this DNA mixture was subsequently used as a template in PCR. The mock community served as a positive control to verify amplification consistency, taxonomic recovery, and sequencing accuracy. After PCR, the mock sample was included in the HTS sequencing run alongside the study samples. Raw high-throughput amplicon sequencing (HTAS) data have been deposited in the NCBI Sequence Read Archive (SRA) under BioProject accession number: PRJNA1330260.

### 2.4. Bioinformatic Analysis

Sanger sequencing data from fungal isolates were quality-checked and visualized in Geneious Prime v2024.0.5 (Biomatters Ltd., Auckland, New Zealand). Taxonomic identification was conducted using the BLASTn (megablast) against the NCBI nucleotide database, with an e-value cutoff of 0.05 and a minimum identity threshold of 98% [36].

HTAS data quality was assessed using FastQC v. 0.12.0 [37]. Read preprocessing and quality filtering were performed in SEED v2.1.2 [38], with paired-end reads merged using fastq-join [39]. Primer sequences and adapters were removed, and sequences shorter than 70 bp or containing ambiguous bases were discarded. Only reads with an average Phred quality score ≥ Q30 were retained. The ITS2 region was extracted from full-length amplicons using ITSx v1.1.2 [40].

Operational Taxonomic Unit (OTU) clustering was performed with USEARCH v8.1.1861 at a 97% sequence similarity threshold [41]. Singleton and chimeric sequences were removed using the UPARSE pipeline [42]. Representative OTU sequences were taxonomically assigned using BLASTn against the UNITE fungal database v8.2 [43], with a minimum identity of 98% and a maximum e-value of 1 × 10^−50^. Non-fungal hits and sequences below the thresholds were excluded [44,45].

### 2.5. Data Analysis

The OTU table was normalized using total sum scaling (TSS) [46]. Community composition was visualized using stacked bar plots and heatmaps generated with matplotlib and seaborn [47]. Alpha diversity was quantified using the Shannon index [48] to capture both species richness and evenness, Simpson’s index to emphasize dominance patterns and the contribution of abundant taxa, and the Chao1 [49] estimator to assess expected species richness by accounting for rare and undetected OTUs, calculated with scikit-bio. Group differences in alpha diversity were tested with the non-parametric Mann–Whitney U test for comparisons among tree species and between sampling years. Beta diversity was evaluated using Bray–Curtis dissimilarity and Jaccard index, with significance tested by PERMANOVA. Principal Coordinates Analysis (PCoA) was performed in scikit-bio, revealing a clear year-based separation of samples. This pattern was independently confirmed by Principal Component Analysis (PCA) of centered log-ratio (CLR) transformed data [46].

Homogeneity of group dispersions was tested using PERMDISP [50], applied to Bray–Curtis and Jaccard distance matrices, to assess differences in within-group variability among species and years. Functional guilds were assigned to fungal OTUs using FUNGuild [51] and grouped into pathotroph, saprotroph, symbiotroph, and mixed categories.

Microbial co-occurrence networks were constructed in Cytoscape v3.10.0 [52]. Relative abundance data were CLR-transformed prior to network inference to account for compositional constraints of sequencing data [46]. Pairwise Spearman correlations were computed, and significant associations (|ρ| > 0.6, *p* < 0.05) were highlighted. Networks were visualized with edge width proportional to correlation strength and node color scaled according to taxonomic or functional category.

All analyses were conducted in Python 3.9 using pandas, numpy, scipy, scikit-bio, seaborn, and matplotlib.

## 3. Results

### 3.1. Sequencing

High-throughput sequencing yielded 20,854,304 reads (read length 40–284 bp). After quality filtering at Q30, 0.01% of reads (2085) were discarded. Reads shorter than 70 bp were removed (additional 4,306,843 reads), leaving 16,545,376 length- and quality-filtered reads. Taxonomic assignment retained 5,819,178 fungal reads for downstream analyses. Clustering at 98% similarity produced 1410 fungal OTUs, of which 319 singletons were excluded, leaving 1091 non-singleton OTUs for analysis. Among these OTUs, 5,076,406 reads (87.2%) could be assigned to the genus level, while 742,396 reads (12.8%) were classified only at the kingdom level. Fungal communities were dominated by Ascomycota with 3,468,779 reads (59.6%), followed by Basidiomycota with 1,607,348 reads (27.6%), Mucoromycota with 268 reads (0.005%), and Glomeromycota with 7 reads (<0.001%). Sequencing depth and data quality were comparable between sampling years.

### 3.2. Relative Abundance

#### 3.2.1. Phylum Level

Across all samples, Ascomycota dominated the wood-inhabiting fungal communities, consistently representing the majority of sequence reads in each host species. Relative abundance ranged from 55–70% in *Fagus sylvatica* ‘Atropunicea’, 70–85% in *Aesculus hippocastanum*, 80–90% in *Quercus robur*, and 80–95% in *Populus alba*. Basidiomycota showed strong host dependence, contributing 25–40% of reads in *Fagus* but only 5–15% in *Quercus* and <10% in *Populus* and *Aesculus*. Minor proportions of Mucoromycota, Chytridiomycota and unclassified taxa (<5% combined) were also detected across hosts. Interannual changes were most pronounced in Basidiomycota, with increased relative abundance in *Fagus* and *Quercus* in 2024 compared to 2023.

#### 3.2.2. Class Level

At the class level, fungal communities were dominated by Sordariomycetes, Eurotiomycetes, Dothideomycetes, Leotiomycetes, and Agaricomycetes, with additional contributions from Tremellomycetes and other minor taxa. Relative abundance patterns were host-specific: in *Quercus robur*, Sordariomycetes accounted for 30–45%, Dothideomycetes for 15–25%, Leotiomycetes for 10–20%, and Eurotiomycetes for 5–15%; in *Populus alba*, Eurotiomycetes comprised 35–50%, Sordariomycetes 20–30%, Leotiomycetes 5–15%, and Dothideomycetes 5–10%; in *Aesculus hippocastanum*, Sordariomycetes represented 20–40%, Eurotiomycetes 15–30%, Dothideomycetes 10–20%, and Leotiomycetes 5–15%; and in *Fagus sylvatica* ‘Atropunicea’, Agaricomycetes reached 20–40%, Tremellomycetes 10–20%, Leotiomycetes 10–15%, Sordariomycetes 5–15%, and Eurotiomycetes 5–10%. Interannual variation was detected primarily in Eurotiomycetes, which increased in *Quercus* in 2024, and in Leotiomycetes, which increased in *Populus* in 2024 relative to 2023.

#### 3.2.3. Order Level

Dominant orders included Pleosporales, Helotiales, Hypocreales, and Eurotiales, with additional contributions from Capnodiales, Chaetothyriales, and Agaricales. In *Fagus*, Agaricales and Polyporales were proportionally higher compared to other hosts, while *Populus* samples frequently contained large fractions of Eurotiales and Helotiales. Interannual variation was reflected in shifts between Pleosporales and Eurotiales dominance in *Quercus* samples.

#### 3.2.4. Family Level

A large proportion of sequences remained unclassified at the family level. Among identified families, Pachysolenaceae, Herpotrichiellaceae, Leucosporidiaceae, and Aspergillaceae were prominent. Nectriaceae, Didymellaceae, and Helotiaceae varied among host species, with Nectriaceae frequently detected in *Quercus* and *Populus*. Some families showed interannual differences, e.g., Didymellaceae were more abundant in *Quercus* in 2023, whereas Helotiaceae increased in 2024.

#### 3.2.5. Genus Level

Among the identified taxa, *Nakazawaea* and *Leucosporidium* were frequent in *Populus*, *Phialophora* and *Pseudocercospora* were recurrent in *Quercus*, and *Ganoderma* and *Pholiota* occurred more prominently in *Fagus*. Potentially pathogenic or endophytic genera such as *Phaeoacremonium*, *Cadophora*, and *Puccinia* appeared sporadically. Year-to-year variation was reflected in a stronger representation of *Leucosporidium* and Helotiales-associated genera in 2024 compared to 2023.

### 3.3. Alfa Diversity

#### 3.3.1. Shannon Diversity Index

**Figure 3:**
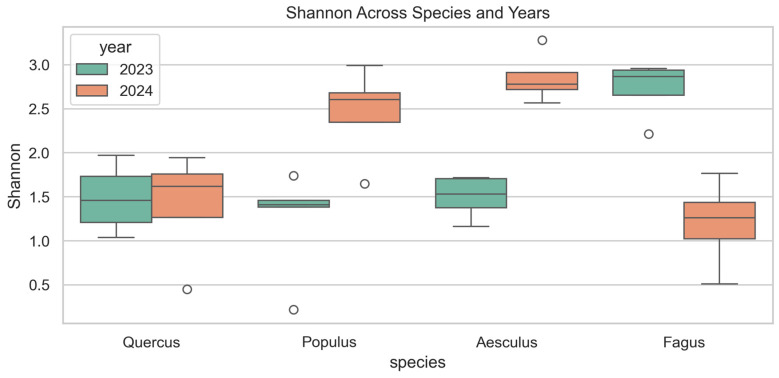
Shannon diversity index of wood-inhabiting fungal communities across four host tree species (*Quercus robur*, *Populus alba*, *Aesculus hippocastanum* and *Fagus sylvatica* ‘Atropunicea’) in 2023 (green) and 2024 (orange). Boxes show median and interquartile range; whiskers indicate data range; circles represent outliers.

Shannon diversity (Figure 3) differed significantly among host species in both years (2023: F = 10.61, *p* = 0.001; 2024: F = 10.00, *p* = 0.001). In 2023, *Fagus* exhibited the highest diversity (median ~ 2.9), followed by *Aesculus* (~1.7), *Quercus* (~1.5), and *Populus* (~1.4). In 2024, *Aesculus* (~2.8) and *Populus* (~2.6) displayed higher values, while *Quercus* (~1.6) and *Fagus* (~1.3) showed lower diversity. Year-to-year comparisons confirmed these shifts: Shannon diversity significantly increased in *Aesculus* (F = 57.37, *p* < 0.001) and *Populus* (F = 12.19, *p* = 0.008), decreased in *Fagus* (F = 23.84, *p* = 0.003), and remained unchanged in *Quercus* (F = 0.04, *p* = 0.856).

#### 3.3.2. Simpson Diversity Index

**Figure 4:**
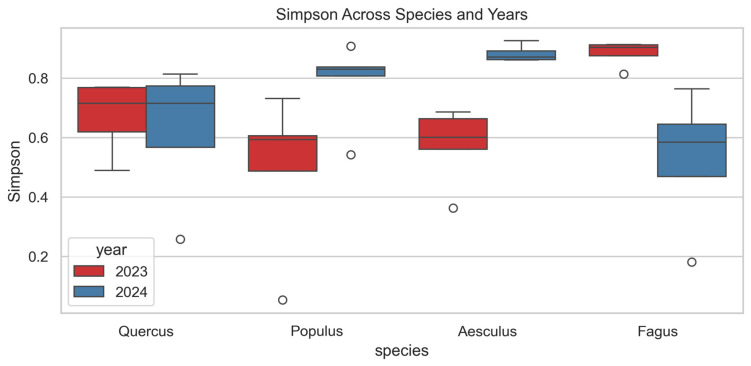
Simpson diversity index of wood-inhabiting fungal communities across four host tree species (*Quercus robur*, *Populus alba*, *Aesculus hippocastanum* and *Fagus sylvatica* ‘Atropunicea’) in 2023 (red) and 2024 (blue). Boxes show median and interquartile range; whiskers indicate data range; circles represent outliers.

Simpson diversity (Figure 4) showed significant species-level differences in 2023 (F = 4.32, *p* = 0.024), but not in 2024 (F = 2.91, *p* = 0.074). In 2023, *Fagus* and *Quercus* reached the highest values (~0.9 and ~0.75), while *Aesculus* and *Populus* showed lower indices (~0.6). In 2024, differences among species were less pronounced, although *Aesculus* and *Populus* tended to exhibit higher values than *Fagus*. Interannual comparisons revealed significant increases in *Aesculus* (F = 21.42, *p* = 0.002) and a decline in *Fagus* (F = 7.95, *p* = 0.030). *Populus* showed a non-significant increasing trend (F = 4.79, *p* = 0.060), while *Quercus* remained stable.

#### 3.3.3. Chao1 Richness

**Figure 5:**
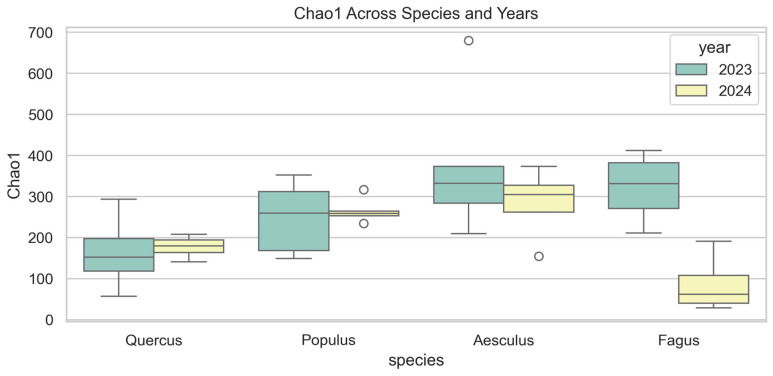
Chao1 estimated richness of wood-inhabiting fungal communities across four host tree species (*Quercus robur*, *Populus alba*, *Aesculus hippocastanum* and *Fagus sylvatica* ‘Atropunicea’) in 2023 (green) and 2024 (yellow). Boxes show median and interquartile range; whiskers indicate data range; circles represent outliers.

Chao1 richness (Figure 5) varied significantly among host species in 2024 (F = 9.32, *p* = 0.001), but not in 2023 (F = 2.43, *p* = 0.108). In 2023, richness was highest in *Fagus* (~360) and *Aesculus* (~330), intermediate in *Populus* (~260), and lowest in *Quercus* (~150). In 2024, *Aesculus* showed the highest values (~310), followed by *Populus* (~260) and *Quercus* (~180), while *Fagus* dropped markedly (~60). Paired year comparisons confirmed a significant decrease in *Fagus* (F = 16.63, *p* = 0.007), while *Quercus* (F = 0.07, *p* = 0.801), *Populus* (F = 0.17, *p* = 0.689), and *Aesculus* (F = 0.83, *p* = 0.392) showed no significant changes.

### 3.4. Beta Diversity

Community composition differed significantly among host species and between years, as confirmed by PERMANOVA (Bray–Curtis: *p* = 0.001; Jaccard: *p* = 0.001).

**Figure 6:**
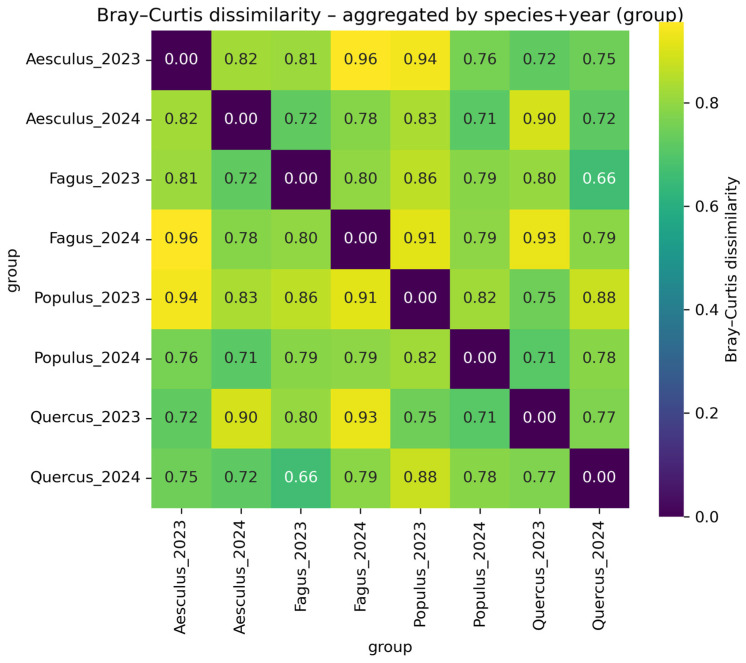
Heatmap of Bray–Curtis dissimilarity aggregated by species and year (group). The heatmap illustrates the Bray–Curtis dissimilarity index values (ranging from 0.0 to 0.96) comparing fungal community compositions across four tree species (*Quercus robur*, *Populus alba*, *Aesculus hippocastanum* and *Fagus sylvatica* ‘Atropunicea’) between 2023 and 2024. Dissimilarity values are represented by a color gradient, where dark purple indicates high similarity (0.0) and yellow indicates higher dissimilarity (up to 0.96).

Heatmap analyses (Figure 6) showed consistently high dissimilarities (Bray–Curtis 0.66–0.96; Jaccard 0.56–0.86). In both metrics, interannual dissimilarities within the same species (e.g., *Fagus*_2023 vs. *Fagus*_2024: Bray–Curtis 0.80; Jaccard 0.85) were comparable to or higher than cross-species dissimilarities within a year. The lowest dissimilarity was observed between *Fagus*_2023 and *Quercus*_2024 (Bray–Curtis 0.66).

**Figure 7:**
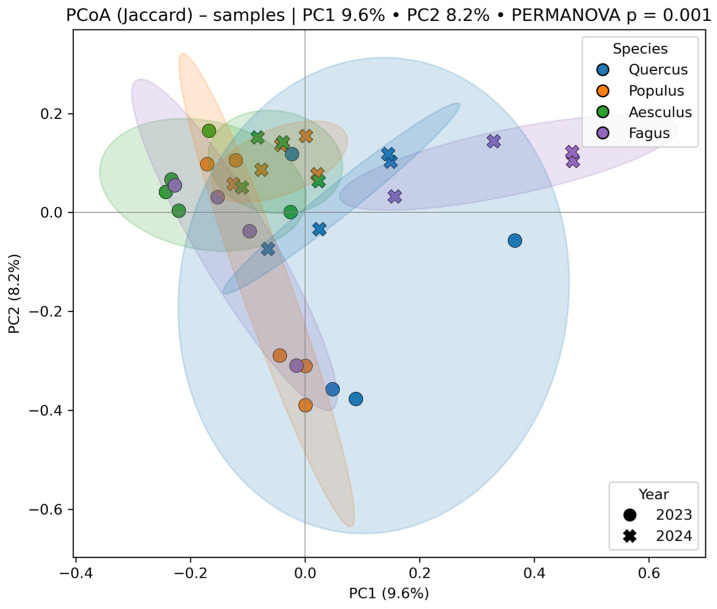
PCoA (Jaccard) of fungal communities associated with four tree species (*Quercus robur*, *Populus alba*, *Aesculus hippocastanum* and *Fagus sylvatica* ‘Atropunicea’) in 2023 (circles) and 2024 (crosses). Ellipses indicate 95% confidence intervals for each species. PERMANOVA shows significant differences among species (*p* = 0.001). PC1 and PC2 explain 9.6% and 8.2% of variation, respectively.

PCoA based on Bray–Curtis distances (PC1 = 12.1%, PC2 = 10.5%) showed partial clustering of samples by host species, with overlaps across groups. Interannual separation was evident for *Fagus* and *Aesculus*. PCoA (Figure 7) using Jaccard distances (PC1 = 9.6%, PC2 = 8.2%) also indicated species-level clustering with distinct year-based shifts. Centroid-based ordination confirmed separation of species × year combinations in multivariate space.

PERMDISP analyses revealed significant differences in dispersion between years for *Fagus* (F = 48.19, *p* = 0.0004), *Populus* (F = 7.21, *p* = 0.0277), and *Aesculus* (F = 9.07, *p* = 0.0196), but not for *Quercus* (F = 0.06, *p* = 0.8108). In *Fagus* and *Populus*, dispersion was higher in 2024 than in 2023, whereas in *Aesculus* it decreased in 2024.

Species-specific PCoA analyses further supported these findings. For *Fagus*, PERMANOVA detected significant year to year differences with Bray–Curtis (*p* = 0.034), but not with Jaccard (*p* = 1.000). *Populus* also showed significant separation between years with Bray–Curtis (*p* = 0.011), but not with Jaccard (*p* = 0.923). *Aesculus* communities differed significantly between 2023 and 2024 based on Bray–Curtis (*p* = 0.035), but not Jaccard (*p* = 1.000). In contrast, *Quercus* did not display significant year-to-year differences in either metric (Bray–Curtis: *p* = 0.328; Jaccard: *p* = 1.000).

### 3.5. Fungal Trophic Groups

**Figure 8:**
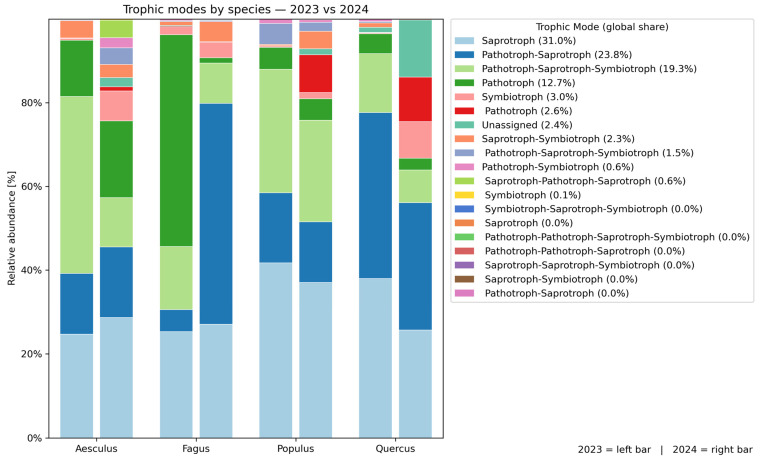
Relative abundance of trophic modes in four tree species (*Quercus robur*, *Populus alba*, *Aesculus hippocastanum* and *Fagus sylvatica* ‘Atropunicea’) in 2023 (left bar) and 2024 (right bar). Colors indicate global percentage of each trophic mode. The plot highlights interannual shifts and host-specific fungal composition.

Functional guild assignment indicated that fungal communities were dominated by saprotrophic and mixed trophic modes across all host species (Figure 8.) When aggregated across species, the largest fractions were saprotrophs (31%), pathotroph–saprotroph (23.8%), and pathotroph–saprotroph–symbiotroph (19.3%), followed by pathotrophs (12.7%). Symbiotrophs accounted for 3% of the community, while unassigned taxa represented 2.4%.

Species-level patterns showed distinct differences. In *Quercus*, pathotroph–saprotroph (35.5%) and saprotrophs (32.5%) dominated, while mixed guilds (11.3%) and pathotrophs (4.7%) contributed smaller shares. *Aesculus* exhibited a broader distribution, with saprotroph–pathotroph–symbiotroph (28.8%), saprotrophs (26.5%), pathotrophs (15.6%), and pathotroph–saprotroph (15.5%) as major groups. In *Fagus*, the most abundant guilds were pathotroph–saprotroph (29%), saprotrophs (26.2%), and pathotrophs (25.9%), together with a notable fraction of pathotroph–saprotroph–symbiotroph (12.4%). *Populus* was characterized by a dominance of saprotrophs (39.4%), followed by pathotroph–saprotroph–symbiotroph (26.9%) and pathotroph–saprotroph (15.6%).

Comparing interannual changes between 2023 and 2024, *Aesculus* exhibited relatively stable functional group proportions between years. In *Fagus*, pronounced changes were observed, with a higher share of pathotrophic and mixed guilds in 2024 and a decrease in purely saprotrophic taxa. *Populus* showed only minor changes, with saprotrophs remaining dominant. In *Quercus*, the 2024 samples contained more pathotrophs and symbiotrophs and fewer saprotrophs, indicating greater ecological variability between years.

At the tree level, interannual comparisons showed variability in functional groupproportions, but saprotrophs and mixed pathotroph–saprotroph modes remained consistently dominant in both years across all host species.

### 3.6. Microbial Co-Occurrence Networks

**Figure 9:**
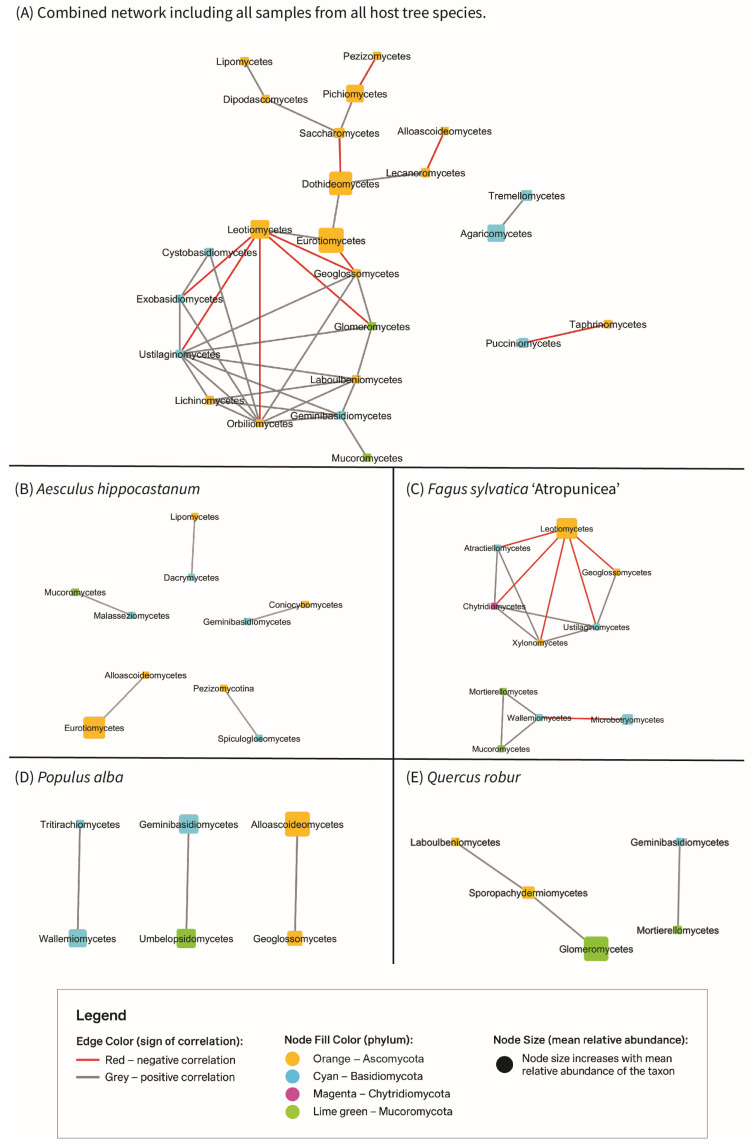
Co-occurrence networks showing associations among fungal classes detected in wood across host tree species. (**A**) Global network including all samples from all hosts. (**B**–**E**) Host-specific subnetworks constructed separately for (**B**) *Aesculus hippocastanum*, (**C**) *Fagus sylvatica* ‘Atropunicea’, (**D**) *Populus alba* and (**E**) *Quercus robur*.

Co-occurrence networks (Figure 9) constructed at the class level revealed distinct interaction patterns across all samples and within individual host tree species. In the overall network combining all samples and years, the most abundant classes formed a highly connected core, with both positive (grey edges) and negative (red edges) correlations. Ascomycota classes dominated the hub structure, particularly with several classes showing multiple strong connections, whereas Basidiomycota and Mucoromycota were represented by fewer, more peripheral nodes. All identified hubs were supported by correlations with *p*-values < 0.01.

In *Aesculus*, the network exhibited a relatively fragmented structure, characterized by fewer highly connected hubs compared to the global network. Most associations were positive, although several negative correlations occurred among dominant Ascomycota classes.

The *Fagus* network was markedly denser, forming a cohesive interaction cluster primarily dominated by Ascomycota. Negative associations were prevalent among the core taxa, whereas Basidiomycota occupied peripheral positions and were connected through only a limited number of edges.

Within *Populus*, the network was partitioned into multiple smaller clusters, with Ascomycota and Basidiomycota representing the majority of nodes. Both positive and negative interactions were detected, yet the overall network cohesion was lower than that observed in *Fagus*.

The *Quercus* network was structured around several abundant Ascomycota classes functioning as hubs, linked by both positive and negative associations. Mucoromycota was present as a minor component, primarily integrated through positive connections.

## 4. Discussion

Wood-inhabiting fungal communities in old-growth trees of Lednice Castle Park showed clear host specificity combined with pronounced temporal dynamics. Fungal richness and beta diversity differed among hosts, while intra-host turnover between years was equally strong, indicating that the wood mycobiome is not static even within mature trees. This temporal instability was most evident in *Fagus sylvatica* ‘Atropunicea’, which exhibited a marked decline in richness and increased beta dispersion in 2024, while *Aesculus hippocastanum* maintained comparatively stable diversity. *Quercus robur* and *Populus alba* showed only moderate interannual change, suggesting that the strength of temporal turnover is host dependent.

Interannual variation was mainly linked to changing proportions of Basidiomycota and to shifts among dominant Ascomycota classes and orders: for instance, Eurotiomycetes increased in *Quercus* in 2024, while Leotiomycetes expanded in *Populus*. Taxonomic composition confirmed strong host filtering. Across all samples, Ascomycota dominated, but *Fagus* harboured a relatively higher proportion of Basidiomycota—especially Agaricomycetes and Polyporales—consistent with its role as a substrate for white-rot fungi and latent wood decayers [53,54]. In contrast, *Quercus* hosted fungal communities strongly dominated by Ascomycota, particularly Dothideomycetes, Sordariomycetes and Leotiomycetes, which are typical for hardwood-associated endophyte assemblages [23,28]. This pattern is consistent with study of Menkis et al. (2022), who reported similar Ascomycota dominance in veteranisation wounds of ~100-year-old living *Quercus robur* trees [55]. *Populus* likewise showed strong enrichment in Ascomycota, a trend widely reported in host-associated mycobiome studies where Sordariomycetes and Dothideomycetes dominate across *Populus* tissues [56,57]. At the genus level, potentially pathogenic or opportunistic taxa such as *Phaeoacremonium* and *Cadophora* were detected sporadically; both are well-documented causal agents of trunk diseases in grapevine, plum, apricot, peach, and various other hardwood hosts [58,59,60,61].

Beta diversity analyses confirmed strong temporal instability [62,63]. Similar patterns of strong temporal turnover, occasionally exceeding host-driven differentiation, have been reported for fungal communities associated with living trees and woody substrates across different forest ecosystems [64]. Consistently, abundance-based (Bray–Curtis) and presence–absence (Jaccard) dissimilarities between years within a host (0.77–0.82; up to 0.85 in *Fagus*) were similar to or greater than interspecific distances. PCoA ordinations showed clear host clustering but strong year-to-year separation in *Fagus* and horse chestnut, while *Quercus* remained relatively stable.

Functional guild analysis revealed a consistent dominance of saprotrophic fungi across hosts, with additional contributions of mixed pathotroph-saprotroph taxa. This pattern matches global findings from wood mycobiome studies [63,65,66]. Mixed trophic guilds represent functionally flexible fungi capable of shifting between endophytic, saprotrophic, and pathogenic lifestyles depending on host condition and resource availability [15,51]. Their high relative abundance therefore indicates a transitional state of the wood mycobiome, in which community structure is reorganized rather than stabilized around a single dominant trophic strategy [16,67]. In *Fagus sylvatica* ‘Atropunicea’, 2024 samples showed a marked increase in pathotrophic and mixed guilds, while saprotrophs declined, indicating stress-driven succession. The high proportion of saprotrophs in living wood most likely reflects latent endophytes that can switch to saprotrophic or pathogenic lifestyles once host defences weaken [68,69]. Such lifestyle plasticity, increasingly recognized in endophytes [15,70,71], explains the guild restructuring observed in *Fagus* and highlights the ecological risk of latent fungi transitioning to opportunistic pathogens under host decline [67].

Co-occurrence network analysis underscored differences in community assembly processes among hosts tree species. The global network was highly connected and dominated by Ascomycota, with both positive and negative associations, while Basidiomycota and Mucoromycota remained peripheral. *Fagus* supported the most cohesive and competitively structured network, characterized by numerous negative correlations that suggest strong intertaxa competition and niche differentiation. Negative associations in fungal networks are commonly interpreted as indicators of competitive exclusion and resource partitioning among co-occurring taxa [72,73]. In wood-inhabiting fungal communities, negative correlations can reflect priority effects, colonization order, and niche partitioning within the heterogeneous woody substrate, rather than direct antagonistic interactions alone [63]. These processes are well documented in fungal ecology and indicate that negative co-occurrence patterns may emerge from indirect interactions and successional dynamics as well as from direct competition among taxa [74]. The balance between positive and negative associations can also serve as a proxy for community stability, where increased negative connectivity often accompanies ecological stress or reorganization [75,76]. In contrast, fungal co-occurrence networks in *Aesculus* and *Populus* were more fragmented and predominantly positive, indicating looser and potentially transient interactions. *Quercus* exhibited well-connected Ascomycota hubs but lacked the dense core observed in *Fagus*, consistent with its more compositionally stable yet functionally flexible mycobiome. A similar dominance of Ascomycota hubs and host-dependent modularity patterns has also been observed across forest types [77]. The network analysis presented here focuses on qualitative structural patterns, whereas quantitative comparison of network metrics was beyond the scope of the present study.

Our results closely align with those of Nordén et al. (2025) [78], who investigated wood-inhabiting fungi in *Quercus*, *Fagus*, *Acer*, and *Tilia* across Norwegian forests. In both studies, saprotrophic fungi dominated the functional structure, while pathotrophic and mixed pathotroph–saprotroph guilds formed a substantial secondary component, suggesting that these functional strategies are conserved across biogeographic regions. Similarly, Ascomycota prevailed over Basidiomycota in both datasets, reflecting the dominance of opportunistic microfungi that frequently colonize early or transitional wood stages. Despite these similarities, our findings also diverge from Nordén et al. (2025) [78] in several key aspects. First, while their study focused exclusively on deadwood, we demonstrate that living and partially decayed wood also hosts taxonomically rich fungal assemblages, including latent pathotrophs typically absent from deadwood surveys. Second, temporal turnover was a major factor structuring communities in our study, whereas Nordén et al. (2025) [78] reported only spatial variation among host trees and bioclimatic zones. Third, we observed strong year-to-year functional shifts, including an increase in pathotrophic guilds under presumed host stress in *Fagus sylvatica* ‘Atropunicea’—a pattern not detectable in static deadwood datasets. These differences indicate that fungal succession inside living trees is driven not only by substrate properties but also by host physiological status and environmental fluctuations.

Overall, our results demonstrate that the wood mycobiome of mature trees is a dynamic and host-dependent system that reflects both internal tree condition and external environmental variability.

## 5. Conclusions

This study demonstrates that the internal wood mycobiome of mature trees is a host-structured but temporally variable system rather than a stable microbial assemblage. Fungal communities were consistently dominated by Ascomycota, yet their taxonomic composition, diversity, and functional guild structure differed among hosts, indicating strong host filtering and species-specific fungal assembly processes. Short-term temporal turnover was pronounced, particularly in *Fagus sylvatica* ‘Atropunicea’, suggesting that fungal community restructuring can occur even within one year.

These findings show that latent shifts in wood-associated fungal communities may precede visible decline, emphasizing the ecological relevance of subcortical fungal dynamics in long-lived trees. They also demonstrate that single-time sampling underestimates fungal variability, reinforcing the need for multi-year monitoring in both ecological studies and tree health diagnostics. Integrating high-throughput amplicon sequencing, beta diversity metrics, and network analysis provides a sensitive framework for detecting early warning signals of internal wood decay and latent pathogen activity, with direct implications for tree health assessment and the conservation management of heritage park trees.

## Figures and Tables

**Table 1:** Overview of sampled trees in the Lednice Castle Park. The table includes tree ID, species, GPS coordinates, estimated age, stem diameter, and stem circumference of each individual.

No.	Species	GPS Coordinates	Tree Age Estimate * [Years]	Stem Diam. ** [cm]	Stem Circumference ** [cm]
1	*Aesculus hippocastanum*	48°47′58.1″ N 16°48′35.6″ E	80–100	79	248
2	*Aesculus hippocastanum*	48°48′49.7″ N 16°48′38.0″ E	80–100	75	236
3	*Aesculus hippocastanum*	48°48′19.1″ N 16°48′32.1″ E	60–80	78	245
4	*Aesculus hippocastanum*	48°48′04.7″ N 16°48′42.3″ E	100–120	111	349
5	*Aesculus hippocastanum*	48°48′12.9″ N 16°48′38.8″ E	100–120	89	280
6	*Fagus sylvatica* ‘Atropunicea’	48°47′56.4″ N 16°48′35.5″ E	≈150	114	358
7	*Fagus sylvatica* ‘Atropunicea’	48°48′00.5″ N 16°48′32.4″ E	150–200	136	426
8	*Fagus sylvatica* ‘Atropunicea’	48°48′03.6″ N 16°48′16.4″ E	≈150	125	393
9	*Fagus sylvatica* ‘Atropunicea’	48°48′10.7″ N 16°48′25.9″ E	≈150	109	343
10	*Fagus sylvatica* ‘Atropunicea’	48°48′47.9″ N 16°48′22.6″ E	150–200	121	381
11	*Populus alba*	48°48′03.4″ N 16°48′48.8″ E	80–100	117	367
12	*Populus alba*	48°48′15.9″ N 16°48′49.2″ E	60–80	87	273
13	*Populus alba*	48°48′19.3″ N 16°49′04.6″ E	60–80	80	251
14	*Populus alba*	48°48′29.9″ N 16°48′47.4″ E	≈100	120	377
15	*Populus alba*	48°48′25.4″ N 16°48′42.0″ E	100–120	133	418
16	*Quercus robur*	48°48′00.7″ N 16°48′50.2″ E	150–200	157	493
17	*Quercus robur*	48°48′40.3″ N 16°48′28.4″ E	150–200	129	405
18	*Quercus robur*	48°48′20.8″ N 16°48′45.4″ E	150–200	153	480
19	*Quercus robur*	48°48′19.8″ N 16°48′38.5″ E	150–200	163	512
20	*Quercus robur*	48°48′48.9″ N 16°48′37.1″ E	150–200	148	465

Note: * Tree age was estimated using a combination of trunk diameter at breast height (DBH) measurements and increment coring. ** measured at DBH.

## Data Availability

Raw high-throughput amplicon sequencing (HTAS) data have been deposited in the NCBI Sequence Read Archive (SRA) under BioProject accession number: PRJNA1330260. All data generated or analysed during this study are included in this published article and Supplementary Material.

## Supplementary Materials

The following supporting information can be downloaded at: https://www.mdpi.com/article/10.3390/jof12010050/s1, File S1: Interactive HTML map displaying the geographic locations of the sampled trees. Each sampling point is annotated with metadata specifying tree ID, species, and GPS coordinates. The file can be opened directly in any standard web browser. Figure S1: Photoplate showing the twenty sampled trees included in this study. The numbering corresponds to individual tree IDs listed in Table 1 and indicated on the map (File S1; Figure 2). Trees 1–5 represent *Aesculus hippocastanum* (horse chestnut), 6–10 *Fagus sylvatica* ‘Atropunicea’ (copper beech), 11–15 *Quercus robur* (pedunculate oak), and 16–20 *Populus alba* (white poplar).

## Abbreviations

DBH	Diameter at Breast Height
HTAS	High-Throughput Amplicon Sequencing
NCBI	National Center for Biotechnology Information
OTU	Operational Taxonomic Unit
PCoA	Principal Coordinates Analysis
PERMANOVA	Permutational Multivariate Analysis of Variance
PERMDISP	Permutational Analysis of Multivariate Dispersion
CLR	Centered Log-Ratio Transformation
TSS	Total Sum Scaling
SRA	Sequence Read Archive
FUNGuild	Fungal Guild Assignment Tool
