# Supplementary material for: Annual Dynamics of Mycobiota in Symptomatic Century-Old Trees of Aesculus hippocastanum, Fagus sylvatica, Populus alba, and Quercus robur
Source: J Fungi (Basel). 2026 Jan 11;12(1):50. doi: 10.3390/jof12010050 (PMC12843164; doi:10.3390/jof12010050)
Supplement: Supplementary file 1 [file jof-12-00050-s001.zip › File S1.html]

**Legend:**  
 *Aesculus hippocastanum*  
 *Fagus sylvatica* ʻAtropuniceaʼ  
 *Populus alba*  
 *Quercus robur*
