# Supplementary figures and images for: Annual Dynamics of Mycobiota in Symptomatic Century-Old Trees of Aesculus hippocastanum, Fagus sylvatica, Populus alba, and Quercus robur
Source: J Fungi (Basel). 2026 Jan 11;12(1):50. doi: 10.3390/jof12010050 (PMC12843164; doi:10.3390/jof12010050)

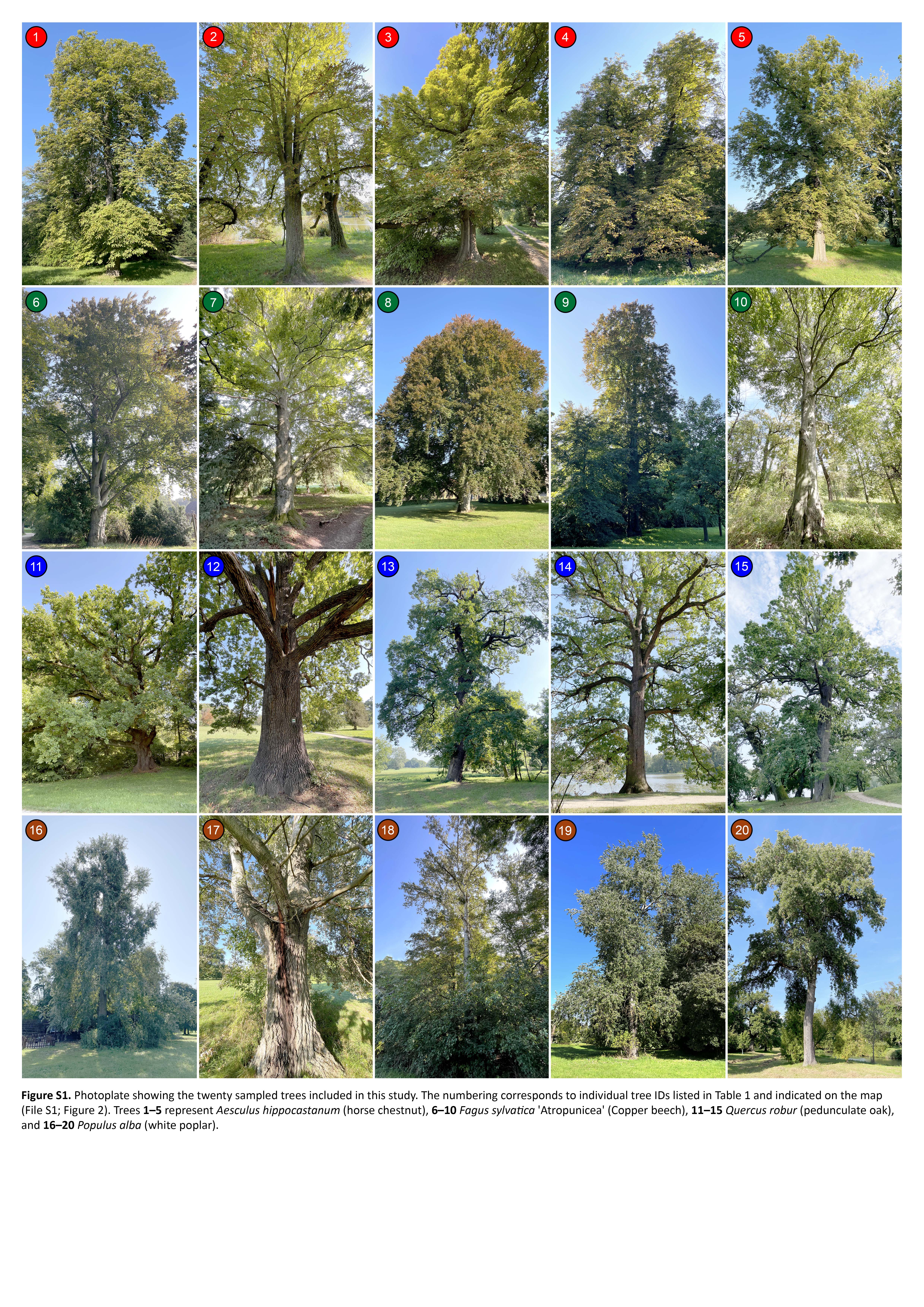

Supplement: Supplementary file 1 [file jof-12-00050-s001.zip › Figure S1.jpg]
